# Synchrotron XFM tomography for elucidating metals and metalloids in hyperaccumulator plants

**DOI:** 10.1093/mtomcs/mfac069

**Published:** 2022-09-13

**Authors:** Kathryn M Spiers, Dennis Brueckner, Jan Garrevoet, Gerald Falkenberg, Antony van der Ent

**Affiliations:** Deutsches Elektronen-Synchrotron DESY, Hamburg, Germany; Deutsches Elektronen-Synchrotron DESY, Hamburg, Germany; Department of Physics, University of Hamburg, Hamburg, Germany; Faculty of Chemistry and Biochemistry, Ruhr-University Bochum, Bochum, Germany; Deutsches Elektronen-Synchrotron DESY, Hamburg, Germany; Deutsches Elektronen-Synchrotron DESY, Hamburg, Germany; Centre for Mined Land Rehabilitation, Sustainable Minerals Institute, The University of Queensland, St Lucia, Australia

**Keywords:** hyperaccumulator, imaging, metal/metalloid, synchrotron, X-ray fluorescence microscopy-computed tomography

## Abstract

Visualizing the endogenous distribution of elements within plant organs affords key insights in the regulation of trace elements in plants. Hyperaccumulators have extreme metal(loid) concentrations in their tissues, which make them useful models for studying metal(loid) homeostasis in plants. X-ray-based methods allow for the nondestructive analysis of most macro and trace elements with low limits of detection. However, observing the internal distributions of elements within plant organs still typically requires destructive sample preparation methods, including sectioning, for synchrotron X-ray fluorescence microscopy (XFM). X-ray fluorescence microscopy-computed tomography (XFM–CT) enables “virtual sectioning” of a sample thereby entirely avoiding artefacts arising from destructive sample preparation. The method can be used on frozen-hydrated samples, as such preserving “life-like” conditions. Absorption and Compton scattering maps obtained from synchrotron XFM–CT offer exquisite detail on structural features that can be used in concert with elemental data to interpret the results. In this article we introduce the technique and use it to reveal the internal distribution of hyperaccumulated elements in hyperaccumulator plant species. XFM–CT can be used to effectively probe the distribution of a range of different elements in plant tissues/organs, which has wide ranging applications across the plant sciences.

## Graphical Abstract

**Graphical Abstract:**
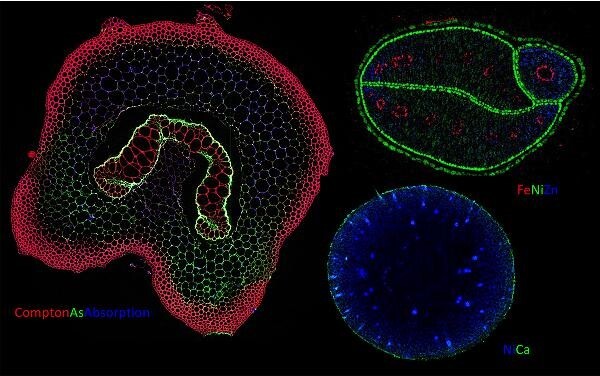
X-ray fluorescence micro-computed tomography reconstructions of various hyperaccumulator plant organs, showing a rachis of Pteris vittata, seed of Noccaea tymphaea and petiole of Pycnandra acuminata.

## Introduction

Metals (and some metalloids) are essential to the functioning of many processes in plants and are highly regulated to counter either deficiency or toxicity. Visualizing the endogenous distribution of elements within plant organs affords key insights in the regulation of trace elements in plants and is essential for a wide range of plant science studies.^1,2^ Methods based on the X-ray fluorescence (XRF) phenomenon are attractive because they are intrinsically nondestructive and able to assess most elements in the periodic table, depending on the sample environment, X-ray source energy, and detector systems. The unique capabilities of the X-ray methods arise from their penetrating nature and the characteristic XRF signatures of the different elements.^3^ Synchrotron X-ray fluorescence microscopy (XFM) is a powerful technique that harnesses XRF to produce elemental images of samples, typically by raster-scanning the sample through a focused X-ray beam and collecting the emitted XRF photons at each scan point. XFM can be used to quantitatively determine element concentrations in plant material attaining typically a spatial resolution of 0.5–2 μm and detection limits down to <10 μg g^–1^ for transition element in areas 10–100 mm^2^.^1,3–6^ X-ray beams are used to excite XRF of a suite of elements of interest, via their K-, L- or M-lines.

### Three-dimensional information by virtual sectioning vs physical sectioning

Synchrotron XFM in two-dimensional (2D) modality by scanning in both lateral directions permits to obtain data on the distribution of elements in whole plant organs, such as leaves, but this necessarily projects all information onto a 2D plane integrating over the depth of the sample. Looking at a leaf, e.g. all the information from the adaxial, abaxial epidermis, and mesophyll are consequently merged on top of each other into a single map. Hence, depth information is lost; to gain this depth information, tissue slices prepared through physical sectioning of the tissues for XFM, are often used to obtain data on the internal distribution within cell types and analytical structurers. However, the preparation of sections of plant organs is fraught with technical challenges that have a detrimental impact on the integrity of the endogenous elemental distribution (as well as chemical speciation). Sectioning of fresh hydrated specimens can lead to element deportment and losses during the sectioning (which is often performed with a vibratome, in which the specimen is immersed in liquid). Chemical fixation of specimens generally causes severe elemental redistribution, and freeze substitution is also problematic with major risks for elemental redistribution.^7,8^ Sectioning of frozen-hydrated specimens using a cryo-microtome followed by freeze drying does fully preserve endogenous element distribution but is extremely challenging to perform properly.^9^ Investigation of sectioned frozen-hydrated specimens is close to ideal, ignoring the problems with sectioning itself, but rather difficult as most synchrotron XFM beamlines use a nitrogen cryostream that can only keep specimens <2 mm frozen, particularly considering the sample is also laterally scanned through the cold nitrogen stream. In addition, mounting of a frozen (very thin) specimen and cryo-transfer of the specimens to the sample scan position under the cryostream is difficult to perform reliably without the specimen thawing and/or developing a layer of ice. Synchrotron XFM experiments with dedicated cryogenic vacuum chamber and cryogenic sample preparation workflows are realized, e.g. at the European Synchrotron Radiation Facility (ESRF), Advanced Photon Source (APS), and Deutsches Elektronen-Synchrotron (DESY).^10–12^

An alternative to destructive specimen preparation methods like physical sectioning is virtual sectioning, or “slice imaging” using computed tomography (CT), which enables “looking into depth” without any modification to the specimen under investigation.^13^ X-ray fluorescence microscopy-computed tomography (XFM–CT) enables reconstruction of “virtual cross-sections” (or a 3D reconstruction from stacked slices or 2D projections) of elemental data from a rotation series of projection images, resulting in reliable studies of trace and bulk elemental distribution while avoiding preparation artefacts.^13–16^ Being chemically or cryogenically fixed or fresh, fully intact specimens (whole plant organs) can be analysed “as is” and the results compete with cryogenically prepared and analysed thin sections.^9^

XFM–CT is reliant on extremely fast and high-throughput XRF detection and processing systems to be practically feasible. The advent of the Maia detector system, which uses a large annular detector geometry to maximize solid-angle, and low-latency, event-mode processing has revolutionized XFM–CT.^17,18^ Maia is designed for event-mode data acquisition, where each detected X-ray event is recorded, tagged by detector number in the array, position in the scan, and other metadata.^19^ New multi-element detectors utilizing similarly fast data readout systems are being deployed, combining multi-element silicon drift detectors (SDD) such as multi-element Vortex detectors, Rococo 4-element detector, or Ardesia with the Xspress3 family (quantum detectors) or FalconX8 (XIA LLC).^12,20–22^ In addition, new SDD with thicker silicon sensor material for improved efficiency at higher energies (20–40 keV) become available, which will extend access to nonoverlapping K-lines of more elements (Mo to La).^19^ Acquisition of single slice XFM–CT can be very fast, comparable to 2D elemental mapping, but the alignment and data analysis is considerably more complicated. Another approach to tomographic probing of specimens is confocal XFM. It allows local 3D probing without the need to measure the complete sample but has the disadvantage of inefficient XRF detection (which may cause radiation damage) and limited depth resolution in the 10 μm range for polycapillaries^23^ and a few micrometers for channel arrays.^24^ Both projection-based XFM–CT and confocal XRF can be extended toward volumetric X-ray absorption near edge structure (XANES) analysis to probe chemical speciation within a specimen (for a case study, see van der Ent *et al.*^25^)

### Limited information depth due to self-absorption

**Fig. 1:**
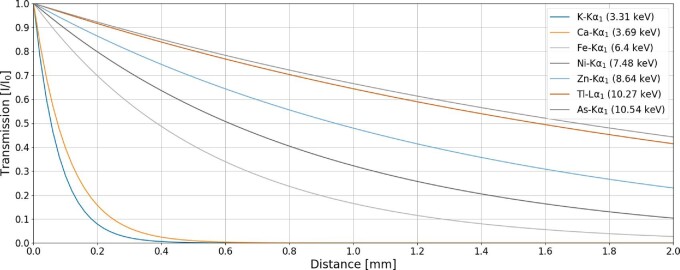
Transmission of selected X ray emission lines through 2 mm of hydrated plant material (with an assumed composition of C7.3O33H59N0.7S0.15) with density 0.9 g/cm^3^. This shows that lower energy characteristic (e.g. Kα1 or Lα1) fluorescence photons emitted from elements such as K or Ca, are absorbed within only 0.2 mm of plant material, whereas higher energy characteristic fluorescence photons emitted from elements such as Tl or As, can pass through more than 2 mm of plant material.

The principal limitation of XFM–CT is self-absorption of escaping fluorescent X-rays, effectively resulting in depth-dependent attenuation (reduction) of the emitted signal, with only the remainder transmitted through the sample to the X-ray detector. The impact of this effect can be readily assessed by examining the absorption tomogram. Attenuation of the incident X-ray beam also takes place, but due to its higher energy this is a less important factor. To illustrate the self-absorption effect, the transmission of emitted fluorescence lines (Kα_1_ or Lα_1_, as relevant) of selected elements through 2 mm of a model hydrated plant matrix (C_7.3_O_33_H_59_N_0.7_S_0.15_) with density 0.9 g/cm^3^, as derived from Wang *et al.*,^26^ is shown in Fig. 1.^27,28^ These transmission data can be used when first estimating whether a localized element within a sample will be detectable. For a sample of 2 mm diameter, 46% of the relatively high-energy 10.54 keV As–Kα_1_ photons would be transmitted the full 2 mm width of the sample, while 68% would transmit through the 1 mm from the center of the sample to the edge. In comparison, essentially none of the K–Kα_1_ or Ca–Kα_1_ photons (3.31 or 3.69 keV, respectively) will be transmitted through these thicknesses; in fact, only 8% of K–Kα_1_ and 15% of Ca–Kα_1_ photons will be transmitted through just 200 μm of this material. It follows that, as long as the same type of fluorescence line is excited (K, L, M) and no absorption edges of high-concentration elements are crossed, the effective thickness of the plant sample (i.e. the longest axis from which fluorescent X-rays can escape) is longer for higher *Z* elements. In practice, most samples are nonuniform in elemental distribution, and this can lead to localized absorption effects. The emitted photons that have been transmitted to the sample surface may then experience further attenuation before they reach the detector (e.g. due to absorption in air), and additionally, for valid detection of each emission line, the sum of photons for that emission line must be above the minimum detection limit (MDL) of the detection system at each analysed point. Thus, for successful measurement, the elemental concentrations in the excited X-ray volume must be high enough to allow for absorption within the sample and through any air path in front of the detector, and then exceed the MDL. The successful detection of elements through a sample using XFM–CT is therefore dependent on a range of factors, from the sample size, and bulk and local composition, the energy and type of emission lines of elements of interest, the experiment setup, and the detection system used.

### Sample selection, preparation, and analytical measurement conditions

Computed tomography yields the best results when projection information is acquired for the full thickness, and with complete angular coverage, of the sample. Large angular spacing or missing angles can result in reconstruction artefacts. An angular range of 180° can provide full angular coverage if self-absorption is negligible, otherwise a 360° angular range should be used. It follows from the previous section that the XFM–CT technique lends itself best to the study of specimens that are “thin” along the two axes perpendicular to the axis of rotation (i.e. approaching a cylindrical cross-section), relative to the absorption of the incident X-ray beam and the relevant absorption lengths of the emission lines of interest emanating from within the sample matrix. A further advantage of thinner samples is that fewer angles are required in a scan for higher resolution angular sampling, resulting in shorter scan times. Therefore, plant organs such as roots and petioles are especially well suited for XFM–CT, but larger complete leaves blades are not. The related technique of “laminography” can be applied to these flatter samples, but will not be discussed further here. The use of hydrated specimens with greater mass density (and consequently higher self-absorption) significantly reduces the size of samples for which XFM–CT is practical, and additionally tends to reduce sensitivity due to the increased scattering from the water matrix. Quantification of XFM–CT is desirable in many applications, but not straightforward due to the complexity of self-absorption effects. Quantification techniques will be discussed in more detail in a following section.

The analytical measurement conditions have the potential to compromise endogenous elemental distribution and coordination chemistry.^9^ It follows that the preparation of specimens must be done in a way that preserves elemental distribution as close as possible to its natural state. The “gold standard” for sample fixation is the use of cryogenic techniques in which the specimens are analysed in frozen-hydrated state as this preserves the distribution, the chemical form, and the concentration of all elements *in situ*.^2,9^ The most used approach involves a liquid nitrogen cryostream system which be placed above the sample to keep its temperature at levels ideally below −140°C (i.e. the vitrification temperature of water) and reduce the influence of beam damage and sample preparation. Plant organs such as roots and rachis, if stiff enough to self-support, can be directly mounted in a holder and for placement in the cold stream of the cryostream, while less stiff samples can be mounted in a Kapton capillary. The formation of an ice layer on the outside of hydrated samples when using a cryostream is a common problem, and efforts should be taken to reduce this phenomenon, as this ice layer can compromise the successful detection of the XRF photons of interest. The ice can cause some absorption of both the incident X-ray beam and emitted fluorescence photons, reducing the detection of the desired signal, and be a source of unwanted Compton scatter, which effectively adds to the noise signal, with both effects contributing to a reduced signal-to-noise ratio. It is also possible to investigate lyophilized specimens, but properly freeze drying these specimens is technically very challenging as it must be performed at a low temperature (starting at −140°C) and proceed very slowly (over several days) to avoid degrading the specimens.^9^ In order to render water in its vitreous form to avoid the formation of potentially destructive ice crystals, freezing of the specimen must happen very fast. The best method is rapid immersion in liquid coolants, such as propane or ethane that are cooled by liquid nitrogen. However, this will only freeze water in vitreous state within the first 10 s of microns into the specimens. Due to their very small thermal mass, specimens will thaw instantaneously when exposed to ambient environment. Cryo-transfer of specimens that are already frozen is practically difficult and time consuming and realized only at a few synchrotron facilities.^10–12^ However, specimens can also be frozen *in situ* under the cryostream. The freezing is rapid (<1 s), although not sufficiently rapid to affect vitreous ice formation.^29^ Diffraction from ice crystals is possible, but has not been observed in our experience in the Maia detector nor disruption of cell walls, leading us to conclude that any ice crystals that had formed were too small to be problematic for the scale of the investigation here.

### The possible impact of radiation damage

The possibility of radiation-induced damage in XFM analysis, especially in fresh hydrated plant tissue samples, is an important consideration that may limit the information sought from the analysis (van der Ent *et al.*^25^) Radiation dose limits for XFM analysis in hydrated plant tissue have been determined to be about 4 kGy before detectable damage occurs, for a 2.5 μm Full Width at Half Maximum (FWHM) focus, oversampled for 2 × 2 μm pixels.^30^ However, samples kept in frozen-hydrated state under a cryostream are immensely radiation hard and did not incur any apparent damage in the elemental maps even at doses as high as 600 kGy. This is highly relevant as the XFM–CT technique exposes the specimen to very high radiation doses.^14,30^

### XFM–CT

**Fig. 2:**
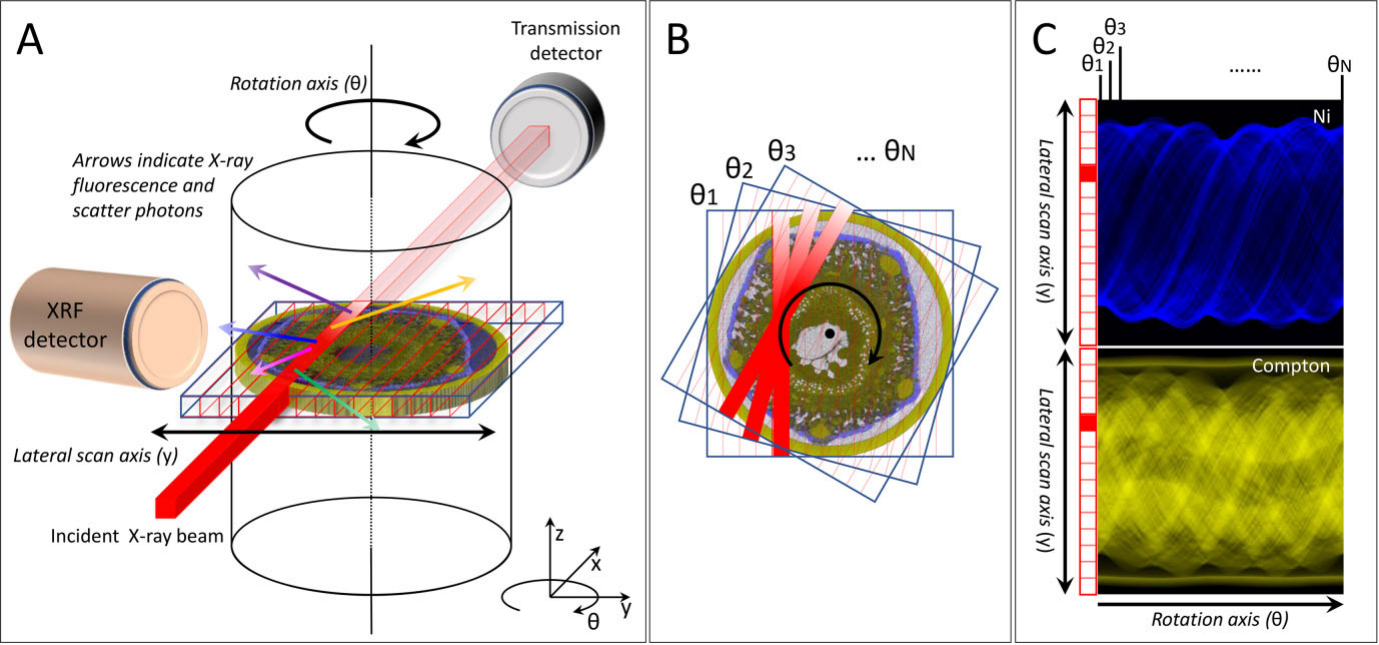
Representation of selected steps in the XFM–CT tomographic process. A: A right-circular cylindrical sample is positioned relative to the incident X-ray beam direction (*x*-axis), the lateral scan (*y*) axis, the vertical (*z*) axis, and the rotation axis (θ). An XRF detector is shown in a typical position, at 90° to the incident beam, and a transmission detector is shown in line with the incident beam behind the sample. The incident X ray beam is illustrated interacting with the sample, causing emission of element-specific X-ray fluorescence photons and scatter (Compton and Rayleigh) photons. B: The progressive compilation of sample scans at several angles, showing the same lateral position as in A. C: Sinogram images produced from fitted spectral data for this *z-*coordinate, with the lateral y coordinate as the vertical axis and the rotation angle (θ) as the horizontal axis. The example shown here is the *Stackhousia tryonii* stem of Fig 9. The Ni fluorescence is shown in blue, and the Compton scatter in yellow. The clear dominance of front side transits (lower left to upper right direction) relative to the back side transits (upper left to lower right direction) indicates a degree of self-absorption in the Ni sinogram. The frozen-hydrated stem was scanned inside a Kapton capillary, which can be observed as the horizontal yellow lines at the top and bottom of the Compton sinogram. The stem cross-section approximates a five-sided shape, with each corner having higher concentration features. These 5 “corners” trace out five brighter sinusoids in the sinograms. The void that is apparent in the center of the sample is also visible as the lighter band in the center of the Compton sinogram. The capillary contains no Ni and is not visible in the Ni sinogram. The example data are reproduced from source data presented in van der Ent *et al.*^31^

Computed tomography is used to reconstruct 2D slices of a sample property, in the case of XFM–CT the elemental distribution, from multiple 1D projections through the sample. Repeated at different heights, it is possible to reconstruct a complete 3D volume of the sample property. The first step of XFM–CT is the acquisition of projected XFM data by scanning the sample through the incident X-ray beam and detecting the emitted XRF photons (Fig. 2A). Defining the beam direction as *x*-direction, the translation of the sample can be performed either in 1D along the *y*-direction (perpendicular to both the incident beam and the axis of rotation) or in 2D in the *yz*-plane (perpendicular to the incident beam). While the former method is faster to yield tomographic results and more resilient to possible interruption of the experiment, the latter is less affected by sample positioning errors in vertical direction. This acquisition process is then repeated for multiple angles by rotating the sample (Fig. 2B). Following the fitting of the energy dispersive XRF spectra using programs like GeoPIXE, PyMCA, or MAPS, the projections for each recorded *z*-coordinate are then sorted into sinograms, resulting in images with the *y*-coordinate as one and the rotation angle as the other axis (Fig. 2C).^32–35^

#### Alignment strategies

Before tomographic reconstruction, the projections may have to be aligned to counteract the effects of instabilities during the measurement, typically a slow drift of the scan center position. If the XFM–CT data were recorded as 2D projections, it is easily possible to correct for vertical misalignment (*z*-direction) before creating the sonograms, e.g. by calculating the cross-correlation between the horizontally integrated projections under different angles. The correlation is then optimized by vertically shifting the projections against each other.^36^ It is important to note that a vertical alignment is not straightforward for 1D projections as they intrinsically lack a vertical component. In those cases, in which vertical drift is to be expected but time constraints prevent the measurement of full 2D projections, it can be beneficial to divide the tomographic scan into multiple overlapping sub-scans with interleaved angles, to better ensure full angular coverage in the case of an equipment failure or similar during the scan. If the vertical misalignment is sufficiently small the sub-scans can be combined or, otherwise, they have to be evaluated separately. There are multiple algorithms to correct horizontal misalignment (*y*-direction) in the projection data. One method for horizontal alignment takes advantage of the fact that the trajectory of single points in a sinogram can be modelled by sine functions. As long as features in the sinogram can be tracked over all recorded angles, the deviation from their ideal sine trajectories can be quantified and corrected.^36,37^ Another method uses the horizontal center of mass of the projections and performs the alignment by shifting the center of mass for each projection to an identical coordinate.^38^ However, the calculation of the center of mass is susceptible to noise in the data and may lead to inaccurate results in cases where all projections have a low signal-to-noise ratio. Finally, the horizontal alignment may be performed using the concept of tomographic consistency. The basic idea of tomographic consistency is the equality of the initial projection and a simulated projection of the reconstructed slice. Differences between measured and simulated projection may be a sign of horizontal misalignment, which can be corrected by iteratively aligning the former to the latter.^39^ One main disadvantage of tomographic consistency is its susceptibility to reconstruction artefacts. As the accuracy of the alignment processes is negatively affected in the presence of self-absorption effects, it is beneficial to perform them either on projections of sufficiently high-energy XRF lines where self-absorption is minimized but not nullified, or, if available, on simultaneously measured absorption projections. The results of those corrections may then be used to align all remaining projections.

#### Tomographic reconstruction algorithms

There are different tomographic reconstruction algorithms to calculate 2D slices from sets of 1D projections and the ideal choice of algorithm is dependent on factors such as data quality, angular sampling, and available computing time. One of these methods is the filtered backprojection (FBP), a single-step algorithm that uses the Fourier transform as well as filters and interpolation in Fourier space to reconstruct the tomographic slice. As a single-step algorithm it is reasonably fast, and it has good resolution characteristics. However, depending on the filter used, it tends to amplify high-frequency noise in the tomogram and is therefore mainly suited for data with good signal-to-noise ratio as well as sufficient angular sampling. Another method is the maximum-likelihood expectation-maximization (MLEM) algorithm, an iterative algorithm that aims to optimize the reconstructed slice in such a way that its projection, using a given tomographic model, is equal to the measured sinogram. It is a slower algorithm compared to FBP but tends to lead to an improved signal-to-noise ratio in the tomograms, albeit at the cost of slightly worse resolution. Increasing the amount of MLEM iterations improves the resolution of the reconstruction but also amplifies any noise in the measurement. The detailed description and mathematical derivation of those algorithms is beyond the scope of this paper. An explanation of some of the more fundamental reconstruction methods can be found in Bruyant.^40^ Many tomographic reconstruction methods are well implemented in established computing libraries such as scikit-image and TomoPy for the Python programming language or the ASTRA Toolbox .^41–43^

#### Quantitative XFM–CT

XFM–CT may also be used as a quantitative method.^15,44–47^ However, depending on the size and composition of the sample as well as the energies of relevant XRF emission lines, it is generally necessary to consider self-absorption effects for quantitative evaluation of XFM–CT data (Fig. 2). Methods used to account for self-absorption effects in XFM–CT include (i) methods using tomographic calibration standards in combination with conventional tomographic reconstruction algorithms, (ii) methods using calibration foils in combination with modified tomographic reconstruction algorithms that account for the self-absorption effects,^44,47^ or (iii) methods utilizing joint reconstructions with the absorption signal collected in standard X-ray transmission (XRT) mode.^45,46^ The first method uses tomographic calibration standards with sizes and elemental compositions ideally as close as possible to the measured samples. In this way the standards show similar self-absorption effects compared to the samples and quantification becomes possible.^14^ This method is a simple one and its main requirement is the time it takes to measure the tomograms of the standards. However, as the standards are only approximations of the samples, this method is not completely accurate. Specifically, it is not suited to correct for reconstruction artefacts arising from self-absorption effects. Alternatively, the self-absorption effects can be accounted for directly in the tomographic model itself.^44,47^ In this case it is sufficient to measure elemental calibration foils and not necessary to measure tomographic standards. Additionally, given the right parameters, this method is fully accurate and also corrects for artefacts caused by self-absorption effects. The downside is the high complexity of the self-absorption problem, leading to slow computation times, as well as the requirement to measure a complimentary absorption tomogram for most implementations. The third method simultaneously collects XRF and XRT tomography data, and combines the signals to solve common unknown variables.^45,46^ The algorithms are typically iterative and ideally provide better self-absorption correction and improved elemental concentration quantification. As with the previously described method, this is computationally heavy.

The basic limitation for all self-absorption correction methods is the signal-to-noise ratio of the measurement. If the self-absorption effects are strong enough that all signal is absorbed and only noise is measured, the correction will only increase the noise.

### Trace element hyperaccumulator plants

Hyperaccumulators are rare plants that accumulate extreme levels of metal and metalloids such as arsenic, nickel, cobalt, copper, zinc, selenium, and thallium in their tissues.^48,49^ Hyperaccumulators are useful models for studying metal(loid) homeostasis in plants because of the extreme expression of constellation of metal(loid) transport and detoxification traits.^9^ Apart from the scientific interest, hyperaccumulator plants are also of considerable interest in potential applications in soil remediation and agromining.^50^ From the perspective of metal(loid) visualization they effectively possess a native contrast agent in the form of the hyperaccumulated element that eliminates the need for extraneous tagging or staining, which in turn has promoted their use as model systems in XFM-based studies.^21,51–53^ The XFM–CT technique has thus far been successfully applied to roots, stems, seeds, and leaves of non-hyperaccumulator plants.^4,5,14,54–56^ XRF and X-ray absorption-edge computed tomography have also been used on hyperaccumulator plants, e.g. to probe Ni in *Alyssum murale* (now classified as *Odontarrhena chalcidica*) and for thallium in *Iberis intermedia* (now classified as *I. linifolia*).^4,57,58^

The aim of this paper is to provide an overview and evaluation of use of the XFM–CT technique applied to plant organs, covering sample preparation, measurement strategies, and data evaluation including tomographic reconstruction. We illustrate the application of synchrotron XFM–CT to in a number of case studies of different organs from hyperaccumulator plants to reveal the internal distribution of hyperaccumulated chemical elements. Plant seeds are ideal subjects for XFM–CT because they are naturally desiccated and often spherical objects in shape. Similarly, fern spores are typically small bodies with low water content that lend themselves particularly well for XFM–CT. Therefore, we showcase the distribution of Zn in a dry seed of *Noccaea caerulescens*, that of Tl in a dry seed of *Biscutella laevigata*, and that of arsenic in dry spores and a freeze-dried rachis of *Pteris vittata*. Investigation of frozen-hydrated specimens is more challenging and will be explored in a frozen-hydrated petiole of *P. acuminata* to reveal Ni-rich laticifer cells, and to reveal Ni in a frozen-hydrated stem of the Ni hyperaccumulator *Stackhousia tryonii*. We hope that these case studies will stimulate plant scientists to consider using XFM–CT in their research.

## Materials and methods

### Synchrotron experiments

**Fig. 3:**
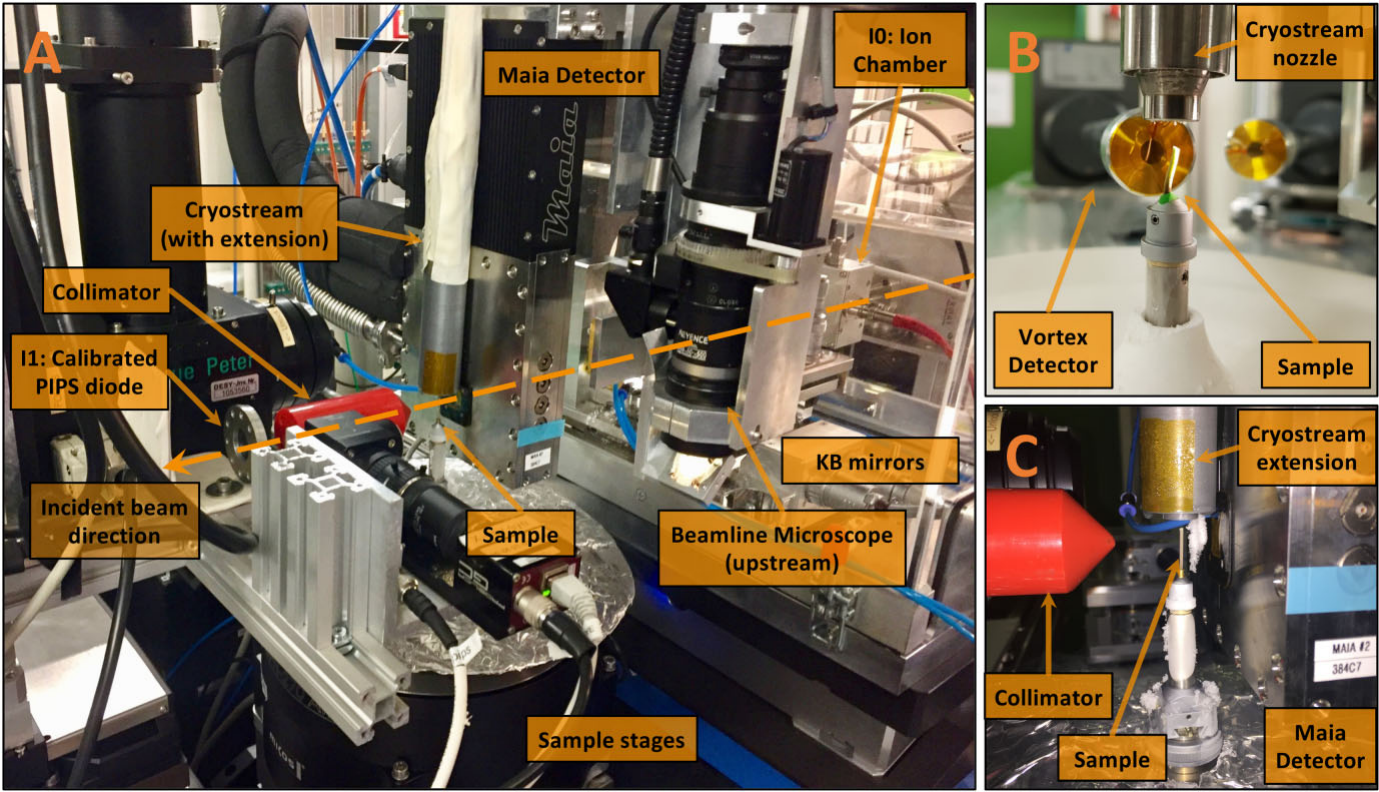
Synchrotron XFM–CT experimental setup at P06 at DESY, with Maia detector and cryostream with extension (A). The (primary) incident X-ray beam passes through an ion chamber (I0) and is then focused by Kirkpatrick–Baez (KB) mirrors before passing through the central opening of the Maia detector to the focus point on the specimen. In an alternative setup (B), the specimen is analysed using a single element detector (Vortex) in a 90° geometry with the specimen (A second single element Vortex detector can be seen in the background; this was unused for these measurements). Free-standing frozen-hydrated petiole under the cryostream, with Vortex detector (B), and specimen held in a Kapton capillary (C). In each case, the specimen is mounted into a holder that is magnetically attached to a rotational stage.

The XFM experiments were undertaken at PETRA III (DESY), a 6 GeV synchrotron radiation source, specifically at the hard X-ray microprobe experiment at the undulator beamline P06.^59^ P06 is equipped with a cryogenically cooled double-crystal monochromator with Si(111) crystals. The X-ray beam was focused to a typically submicron spot using either a Kirkpatrick–Baez (KB) mirror pair (JTEC, Japan) or a stack of 44 Beryllium compound refractive lenses (CRLs) (RXOPTICS, Germany) at incident X-ray energies of 12, 14, or 15 keV with photon flux in the beam focus in the order of 10^10^ photons s^−1^. A Si PIPS diode 500 μm thick and with 19 mm diameter active area (PD300-500CB, Mirion Technologies (Canberra) GmbH, Germany) was located downstream of the sample to record the transmitted X-ray intensity in order to extract absorption data. The incoming flux was monitored by an ionization chamber upstream of the focusing optics. XRF detection was performed by either a Maia 384C detector^17,18^ located upstream from the sample in backscatter geometry or a 50 mm^2^ SII Vortex EM Si-drift detector (Hitachi High-Tech, USA) in 90° geometry (Fig. 3). The tomographic measurements were conducted using 1D (single slice) as well as 2D projections. Scan parameters are detailed in Table 1.

**Table 1.** Experimental and scanning parameters for the different tomography experiments

	Fig. 4A	Fig. 4B, C	Fig. 5A	Fig. 5B–G	Fig. 6A	Fig. 6B	Fig. 6C, D	Fig. 7	Fig. 8	Fig. 9
**Species**	*Biscutella laevigata*	*B. laevigata*	*Noccaea tymphaea*	*N. tymphaea*	*Pteris vittata*	*P. vittata*	*P. vittata*	*P. vittata*	*P*ycnandra* acuminata*	Stackhousia *tryonii*
Plant organ	Seed	Seed	Seed	Seed	Pinna + spores	Pinna + spores	Pinna + spores	Rachis	Petiole	Stem
Detector	Maia	Maia	Maia	Maia	Maia	Maia	Maia	Maia	Vortex	Maia
Focusing optics	Kirkpatrick–Baez (KB)	KB	KB	KB	KB	KB	KB	KB	CRLs	KB
Beam size (nm × nm)	400 × 450	400 × 450	400 × 450	400 × 450	1030 × 650	1030 × 650	1030 × 650	540 × 450	500 × 500	540 × 450
Beam energy (keV)	15	15	15	15	14	14	14	14	12	14
Scan axes^a^	*y, z*	*y*, θ	*y, z*	*y*, θ	*y, z*	*y*, θ	*y, z*, θ	*y*, θ	*y*, θ	*y*, θ
No. lateral steps^b^	2500 × 2500	1250	1290 × 2250	720	1400 × 1400	1400	1400 × 1400	4000	1000	2600
Step size (μm)^c^	1	2	2	2	1	1	5	0.5	2	0.5
Angles^d^		452		452		401	41	1808	452	1804
Sub-scans^d^		2		2		1	1	8	2	4
Dwell time (ms)	1	1	2	1	1	1	1	10	4	7
Slices^e^		46		51		1	281 (proj.)	1	22	1
Slice distance (μm)^e^		48		40			5 (proj.)		5	
Cryostream	No	No	No	No	No	No	no	Yes	Yes	Yes
Presentation	In air	In air	In air	In air	Capillary	Capillary	Capillary	In air	In air	Capillary
Sample preparation	Naturally dried	Naturally dried	Naturally dried	Naturally dried	Freeze dried	Freeze dried	Freeze dried	Freeze dried	Frozen hydrated	Frozen hydrated

^a^Scan axes: 2D data collected for *y, z* (2D planar) or *y, θ* (single slice tomography) axes, 3D data for *y, z, θ*.(see axes, Fig. 2).

^b^No. lateral steps: Corresponds to *y*- or *z*-axes indicated in scan axes. The images shown may be cropped.

^c^Step size: May be under- or over-sampled, i.e. larger or smaller than beam size.

^d^Angles, sub-scans: Indicates total angles scanned; these may be in overlapping sub-scans of interleaved angles.

^e^Slices, slice distance: indicates where multiple single-slice tomograms were collected, and distance between them in the z-axis. Not all are shown.

The specimens were mounted inside Kapton capillaries (*Stackhousia, Pteris*) or glued on top of a pin (*Biscutella, Noccaea* seeds) to allow for easy tomographic measurement. The *Pycnandra* and *Stackhousia* specimens were analysed in frozen-hydrated state under the cryostream while the other specimens were dried and analysed at room temperature. *Stackhousia* was measured in single-slice tomography mode, as were *Biscutella* and *Noccaea*, which in addition were also measured at different sample heights (multiple single-slice tomography). *Pteris* was measured in 2D-projection-based tomography mode. The detailed scanning parameters are provided in full in Table 1.

### Data processing and statistics

The XRF event stream was analysed either using the dynamic analysis method as implemented in GeoPIXE or nonlinear least-squares fitting as implemented in PyMCA.^33,34,60,61^ The tomographic data were, as far as possible, aligned using cross-correlation (vertical direction) and tomographic consistency (horizontal direction) methods to correct for unwanted sample movement. The abundant angular sampling and statistics of the tomographic data of the *Stackhousia* sample allowed to use the FBP method for reconstruction, while the other sparser datasets were reconstructed using an MLEM algorithm. All tomograms shown in this paper were analysed in a qualitative manner as the targets of studies were elemental distribution only, omitting quantitative aspects for efficiency of the measurement series. Since the analysis of these hyperaccumulator plants is a multi-element problem, with distribution of the elements the main focus, and as self-absorption effects did not have a limiting influence on the visualization of the distribution of most of the hyperaccumulated elements of interest, no self-absorption corrections were performed. Of note, self-absorption can be observed in Fig. 8, but this does not, in this case, prohibit visualization of the hyperaccumulated element in the cells of interest. The 3D rendering of the *Pteris* tomogram was performed using the open-source visualization software Drishti.^62^

## Results

### Thallium in dry seed of *B. laevigata*

**Fig. 4:**
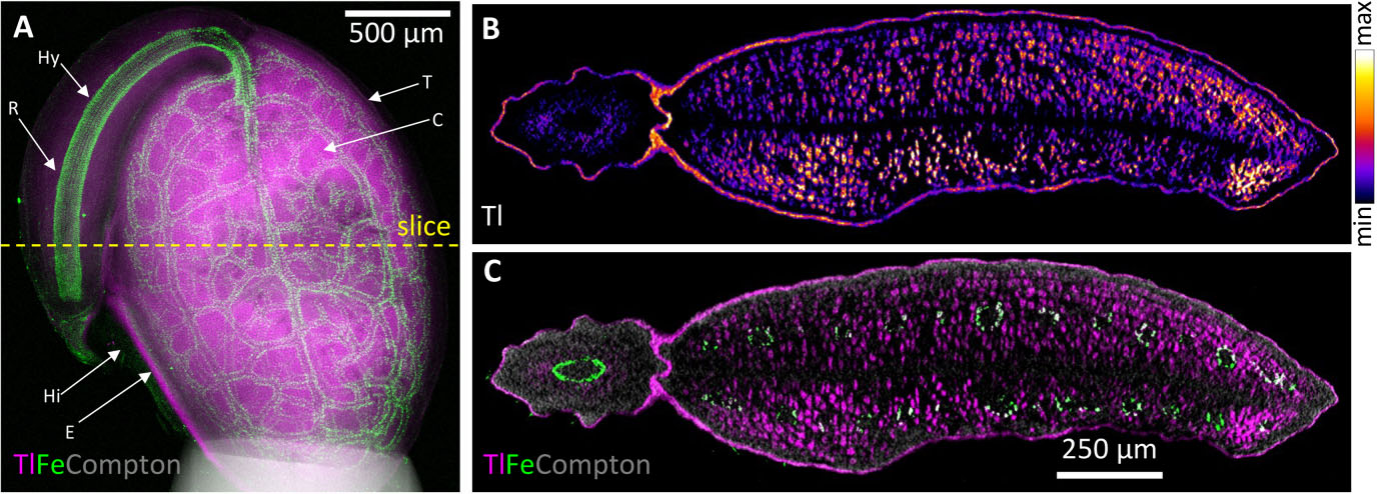
Synchrotron XFM 2D projection map (A) and XFM–CT reconstruction (B, C) of Fe, Tl, and Compton in a *Biscutella laevigata* seed. The projection composite view of the seed (A) shows a superposition of Tl (magenta) and Fe (green) in internal structures with the brightest feature in the Compton (gray) being the glue at the bottom of the seed. The position from which the tomographic slice in B, C was taken is indicated. The relative elemental concentration of Tl is indicated with brighter shades denoting higher prevailing concentrations (B). A composite image of Tl (magenta) and Fe (green) superimposed on the Compton image (gray) shows the spatial relationship between Tl, Fe, and the seed structure, represented by the Compton signal (C). T, testa; Hi, hilum; E, endosperm; R, radicle; Hy, hypocotyl; and C, cotyledon. Reanalysed from source data presented in Corzo Remigio *et al.*^65^

Thallium hyperaccumulation is recognized at the threshold value of 100 μg g^−1^ Tl in shoots and the strongest known Tl hyperaccumulator plant species is *B. laevigata* is capable of attaining up to 32 000 μg g^−^^1^ Tl in its leaves whole growing in soils with 427 μg g^-1^ Tl median concentration.^48,63^ Thallium is readily taken up from the soil by most plants because Tl is generally present as a thermodynamically stable monovalent ion, with similar chemical and solubility properties as monovalent K^+^.^64^ Synchrotron XFM has shown that Tl is localized mainly in the foliar veins of another Tl hyperaccumulator, *Iberis linifolia*, in the form of Tl^+^.^57,58^ A planar 2D projection view of the seed of *B. laevigata* (Fig. 4A) shows that Tl is distributed mainly throughout the endosperm and the vacuoles of the mesophyll cells of the cotyledons (seed leaves), while it is also enriched in the testa (seed coat) but depleted in the vascular bundles. Iron enrichment is observed in the vascular tissue of the radicle and in the vascular bundles in the seed. However, this projection view is a superposition of all internal structures, and, e.g. superimposes the cotyledons. Complementing this projection with the XFM–CT reconstruction of a single-slice tomogram, selected from a set of multiple slices, through the seed (Fig. 4B, C) provides additional information, in particular identifying the Fe vasculature in the two cotyledons and revealing nonuniformities in the Tl distribution in the testa and cotyledons.^65^ The self-absorption of the relatively low-energy potassium K-fluorescence was too high to allow a tomographic reconstruction for comparison with the localization of Tl. The elliptical aspect ratio of this seed, with dimensions of approximately 2000 × 500 μm^2^ at its widest point, is inefficient for tomography, as only the central 500 μm of the 2500 μm lateral scan contains “occupied” pixels when this narrow aspect is presented to the beam. The implementation of advanced tomographic scanning algorithms may allow dynamic adaption to sample width, while still maintaining sufficient information for analytical alignment and reconstruction.

### Nickel and zinc in a seed of *Noccaea tymphaea*

**Fig. 5:**
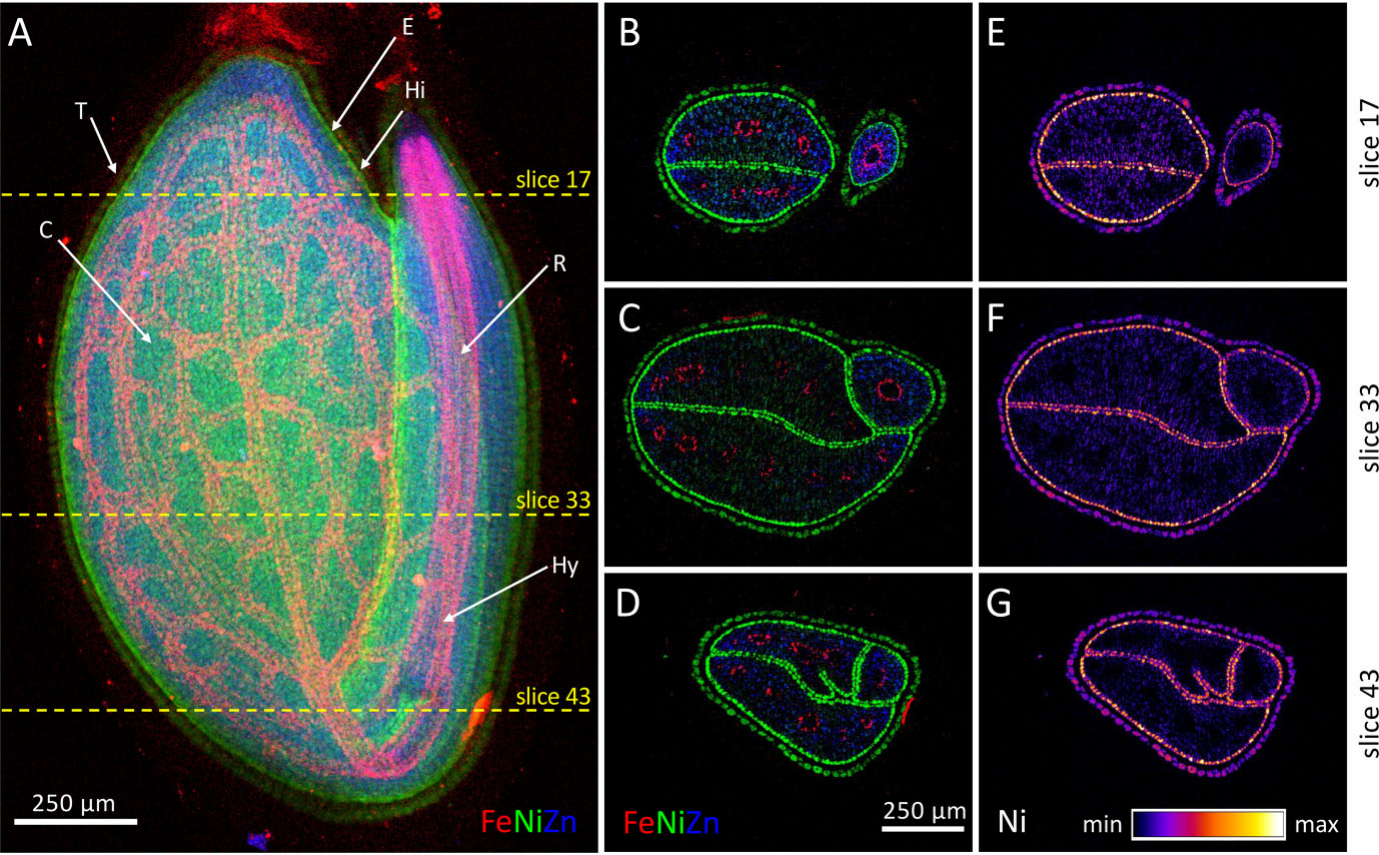
Synchrotron 2D projection map (A) and XFM–CT reconstructions (B–G) of a *Noccaea caerulescens* seed. A total of 51 tomographic slices were collected through the height of the seed; the selected XFM–CT reconstructions for slices 17, 33, and 43 show the distribution of Fe, Ni and Zn (B–D) and Ni intensity (E–G) T, testa, Hi, hilum, E, endosperm, R, radicle, Hy, hypocotyl, C, cotyledon. Reanalysed from source data presented in van der Ent *et al.*^72^

The genus *Noccaea* is unique in that it has over 20 taxa that hyperaccumulate a range of transition elements, including Ni, Zn, Cd, and Pb, with several species hyperaccumulating more than one element simultaneously.^66,67^ This species is unique in that it possesses calamine, ultramafic, and non-metallicolous ecotypes, each represented by different accessions, which differ in metal tolerance and hyperaccumulation abilities. The polymetallic Ni, Zn Cd hyperaccumulator *N. caerulescens* is the most intensively studied hyperaccumulator plant species.^68–70^ Another related species in *N. tymphaea* occurs on ultramafic soils and accumulate high foliar Ni with up to 11 800 μg g^−^^1^ Ni) and relatively low foliar Zn with up to 179 μg g^−^^1^ Zn.^71^ A planar 2D projection of a *N. tymphaea* seed (Fig. 5A) combining Fe, Ni, and Zn shows Ni and Zn distributed throughout the seed, with some resolution of Ni in the testa. Fe enrichment can be clearly observed in the vascular tissue of the hypocotyl and radicle and in the vascular bundles in the cotyledons. However, in this projection, it is not possible to discern further localizations, particularly of Ni and Zn. A set of 51 single-slice tomograms at 40 μm spacing were collected across 2 mm of this seed, and three slices were selected at positions indicated (Fig. 5A), with the corresponding Fe, Ni, and Zn (Fig. 5B–D) and Ni relative concentrations (Fig. 5E–G) revealing elemental localization information that the projection view was unable to discern. Slice 17 shows the hypocotyl separated from the cotyledons, slice 33 shows where the hypocotyl and cotyledons join, and slice 43 shows part of the radicle folded up inside one of the cotyledons. Nickel is localized in the epidermal cells of the cotyledons and radicle (and also the testa), while Zn is more diffusely distributed in the cotyledon blades.^72^ The characteristic enrichment of Fe is observed in the provascular strands of the hypocotyl and radicle, and vasculature of the cotyledons is clearly visible.^72^

### 3.3 Arsenic in freeze-dried spores and rachis of *P. vittata*

**Fig. 6:**
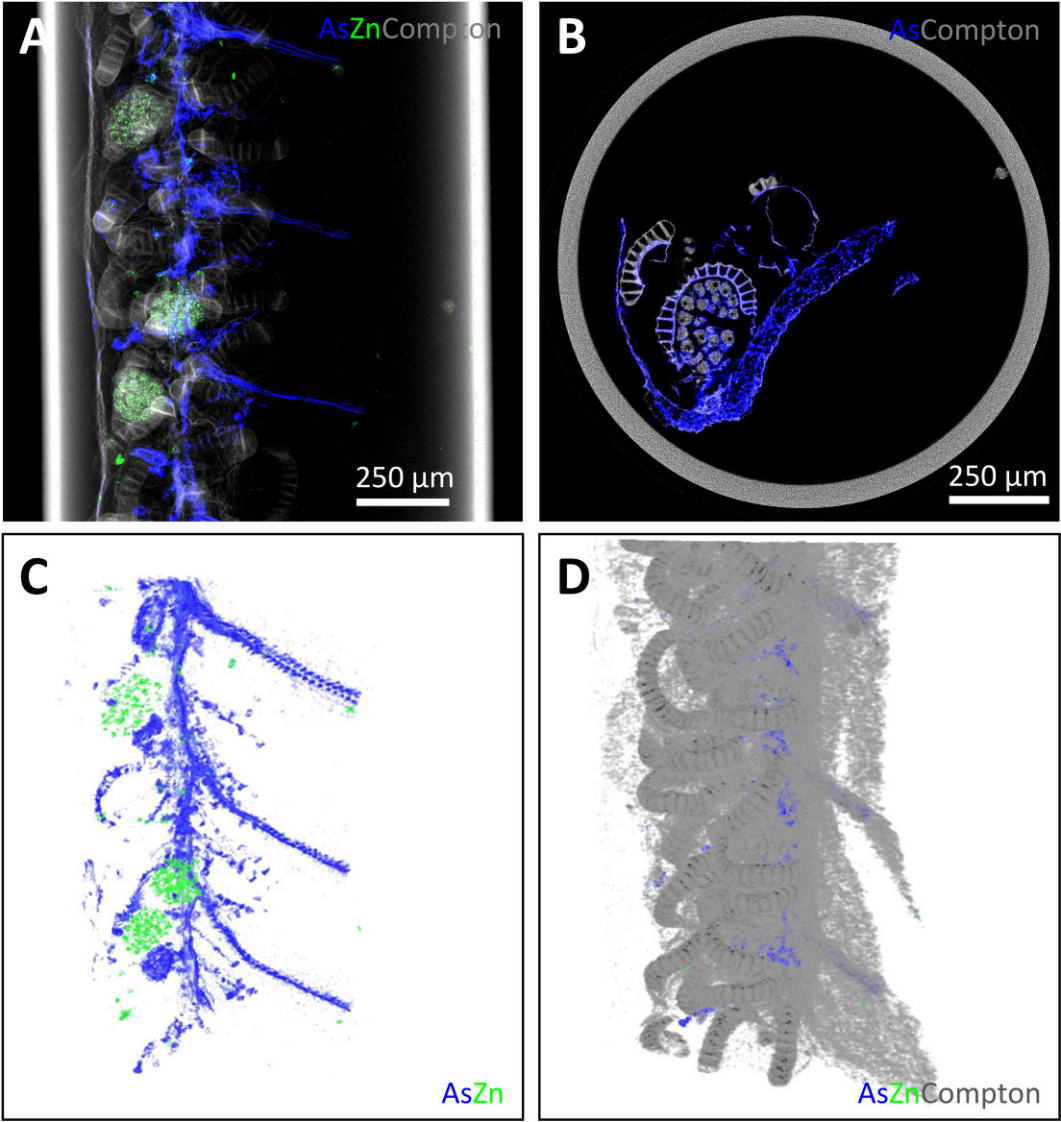
Synchrotron XFM scans of freeze-dried *Pteris vittata* pinna margin. A: 2D projection shows As (blue) predominantly in vascular bundles, and Zn (green) in sporangia (spore cases) clustered into sori, with these signals superimposed on the Compton image (gray). B: Single-slice XFM–CT reconstruction showing Compton (gray) and As (blue), revealing the lateral extent of the vascular bundles relative to the sori. C, D: Frames from within a rendered XFM–CT full 3D dataset of the same region in B, with the overall structure represented by the Compton signal, and As in vasculature and Zn in sori. (See Supplementary Information for video of full rendering). Reanalysed from source data presented in van der Ent *et al.*^25^

**Fig. 7:**
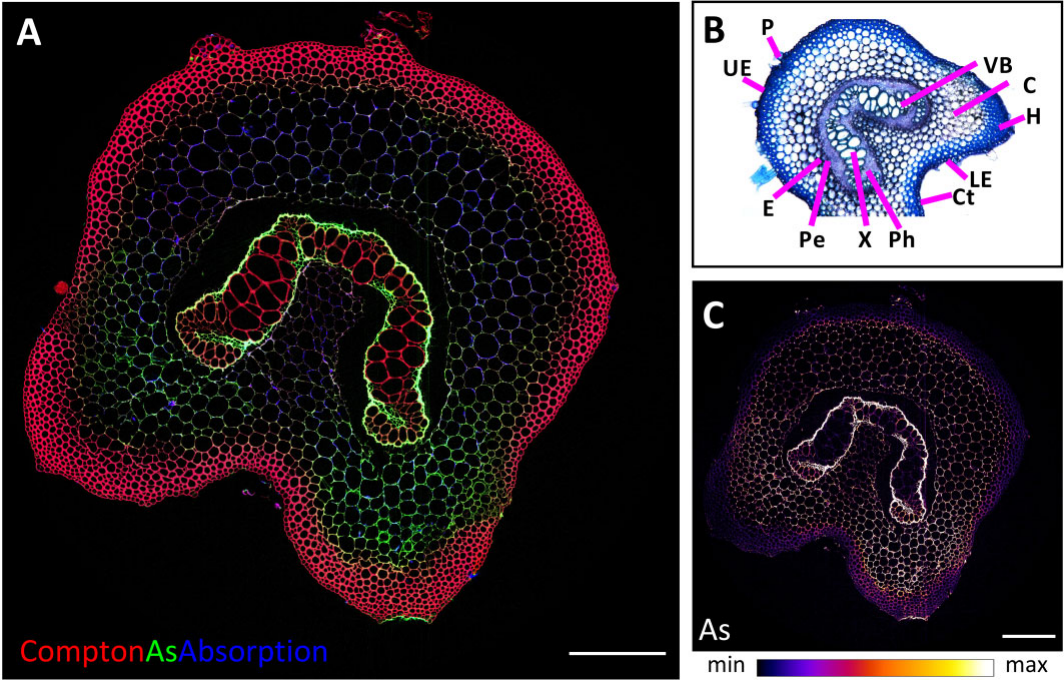
Synchrotron XFM–CT reconstruction of the distribution of As, Compton scatter and absorption in freeze-dried *Pteris vittata* rachis (A). The colors denote relative elemental concentrations with brighter shades denoting higher prevailing concentrations. Toluidine blue stained *P*. *vittata* section of the rachis (B); Abbreviations: LE, lower epidermis; UE, upper epidermis; C, cortex; P, pamenta; VB, vascular bundle; Ct, cuticle; H, hypodermis; X, xylem; E, endodermis; Pe, pericycle; and Ph, phloem. As intensity (C). Reanalysed/reproduced from source data presented in van der Ent *et al.*^25^

The tropical fern *P. vittata* (Pteridaceae) can accumulate up to 22 630 μg g^–1^ As in its fronds.^73^ An XFM 2D projection scan (Fig. 6A) of As, Zn, and the Compton signal and XFM–CT single-slice tomographic reconstruction of As and Compton (Fig. 6B) reveals details within a freeze-dried pinna margin (the edge of a primary-division frond) containing the indusium covering the sporangia (spore cases). The As is enriched predominantly in the long vascular bundles connecting to the sporangia, but little in the sporangia themselves, while Zn is highly localized in sporangia clustered within the spheroidal bounds of mature sori.^25^ The Compton scatter map is very useful in showing the structural features of the tissues to provide context to the distribution of As, Zn, and other elements. Selected frames from a rendering of a full 3D dataset comprising a series of 2D projections without (Fig. 6C) and with the Compton signal (Fig. 6D), firstly reveal the localizations of As and Zn, and then show how these elements contained within other structural material (defined by the Compton signal) in the pinna (see Supplementary Information for video of full rendering). The examination of this sample, with its asymmetries and elements localized to very specific regions (organs) demonstrates both the strengths and limitations of complementary 2D datasets (projection and single-slice tomography) in comparison to a full 3D tomographic reconstruction. While the two 2D image sets can be used to infer the 3D information shown in that dataset, the rendering of the full 3D dataset is visually clearer and richer in mineable data. Conversely, the 2D datasets are faster to collect, require less post-collection analysis and can often supply the necessary information for adequate elucidation of the desired sample characteristics. The XFM–CT reconstructions of a freeze-dried rachis from *P. vittata* (Fig. 7A, C) indicates the distribution of As within the rachis virtual cross-section. The Compton signal typically shows sensitivity to light elements, and clearly dominates structural definition within the hypodermis and xylem, while stronger absorption can be observed in the cortex, a region also relatively high in As. As is enriched in the endodermis and pericycle surrounding the vascular bundles^25^ with the As in the cortex more concentrated toward the lower epithelium, the lower side of the rachis. The freeze drying has apparently caused some shrinkage of the inner structures, and any water soluble As compounds would have precipitated into cell walls.

### Nickel in a frozen-hydrated *P. acuminata* petiole

**Fig. 8:**
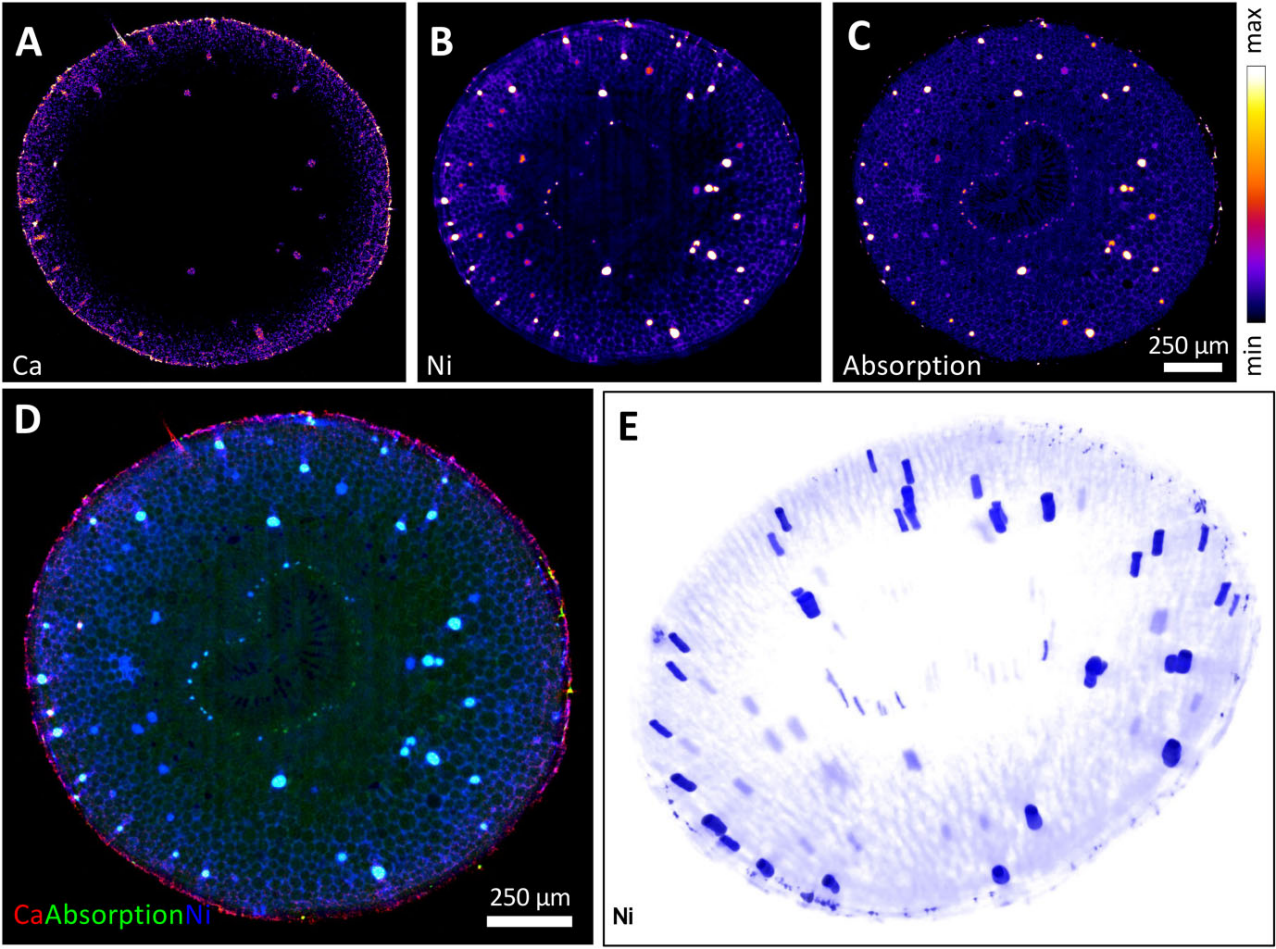
Synchrotron XFM–CT single slice tomographic maps of the distribution of Ca, Ni, and absorption signal (A, B, C) and merged (D), and a 3D rendered view of Ni distribution from 22 single tomographic slices (E) in a frozen-hydrated *Pycnandra acuminata* petiole. Self-absorption can be seen in the Ca image from ∼100 μm into the sample, and in the Ni image from ∼300 μm into the image.

The New Caledonian endemic tree *P. acuminata* has a vivid blue–green latex that contained 25 wt% Ni. The extraordinary concentration of nickel within *P. acuminata* laticifers functions as an effective natural tracer for XFM–CT to probe the structure and organization of these cells, as demonstrated in a XFM–CT reconstructed 2D slice of a petiole (Fig. 8). Ni-rich laticifer cells are clearly indicated by the highest concentration in the Ni map (B) with lower intensity Ni signals defining other structures. Consideration of Ca (3.69 keV) and Ni (7.48 keV) fluorescence and the absorption of the incident 12 keV X-ray beam, and the corresponding reconstructed maps of the 1.5–1.6 mm diameter petiole demonstrate the combined effects of self-absorption and detection limits on the elucidation of different signals. Ca fluorescence is detected from likely Ca-oxalate deposits, which might be expected through the thickness of the sample, only on the surface and to a depth of about 150–200 μm, and higher concentration deposits co-located within some laticifer cells are detectable to a depth of about 400 μm. The Ni and absorption maps are generally visually well correlated, unsurprisingly since higher absorption should be expected from the highly concentrated Ni. However, at about 600 μ@m into the sample, some smaller, lower-intensity laticifers are clearly visible in the absorption map but not in the Ni maps. The reduced detection of Ca and Ni at these lower concentrations and greater depths corresponds to absorption of these fluorescence signals such that the resultant signal falls below the detection limit of the fluorescence detector (Fig. 1). The higher energy incident X-ray beam suffers less absorption through this sample and hence the transmitted signal conveys information, albeit element nonspecific, that is lost from the lower energy fluorescence signals. The 3D rendering of multiple single-slice tomograms (Fig. 7E) shows variations in the structure of the laticifers including variations in diameter and relative concentration of Ni.

### Nickel in a frozen-hydrated *S. tryonii* stem

**Fig. 9:**
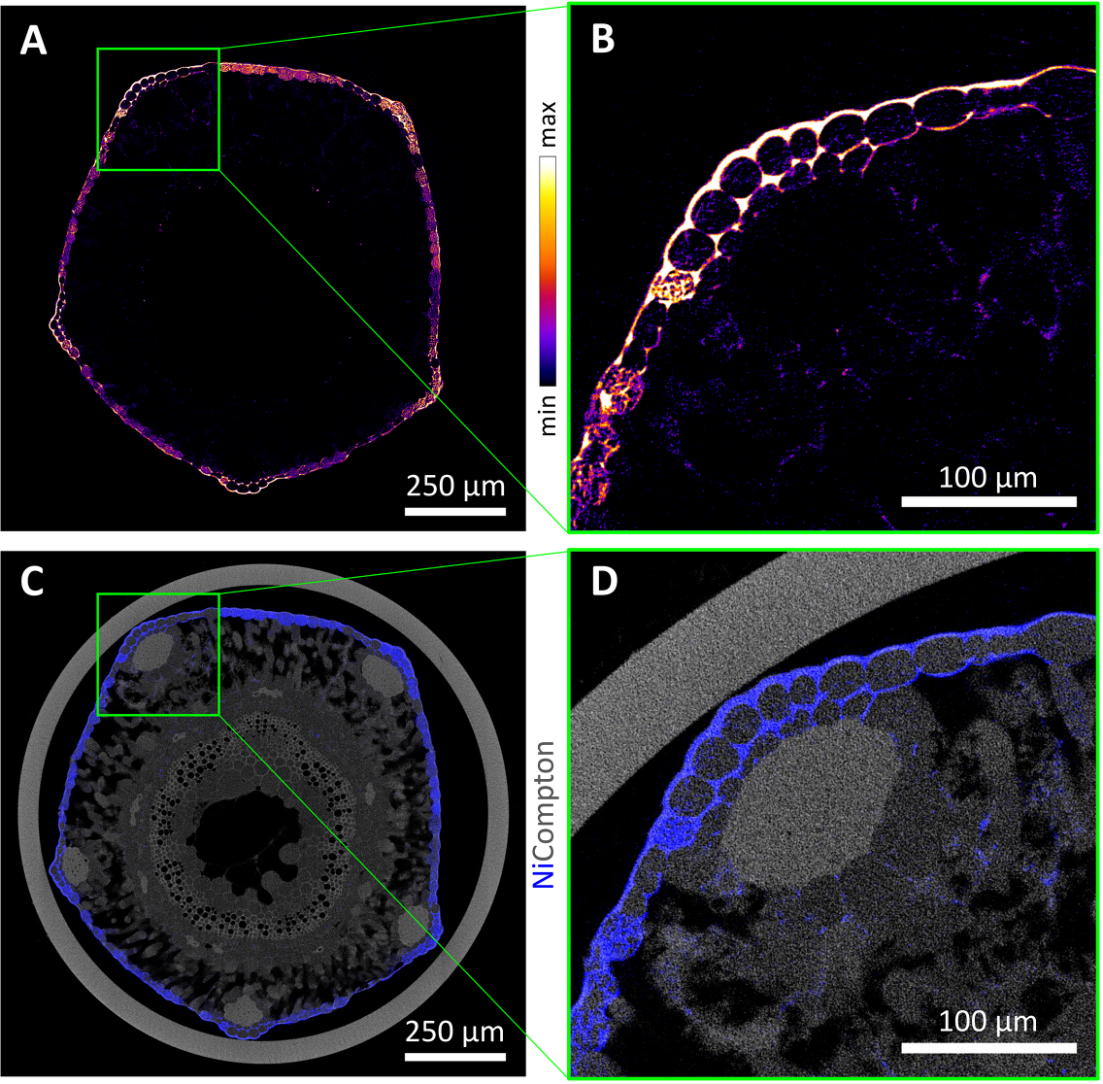
Synchrotron XFM 2D maps of the distribution of (A) Ni, showing relative intensity, and (B) inset, showing that Ni is mainly concentrated in the apoplastic space surrounding epidermal cells (C, D) Ni (blue) and Compton (gray), showing Ni distribution relative to internal structures, in a frozen-hydrated *Stackhousia tryonii* stem. Reanalysed/reproduced from source data presented in van der Ent *et al.*^31^

The perennial herb *S. tryonii* (Celastraceae) from Queensland, Australia can accumulate Ni up to 41 300 μg g^–1^ in the leaves.^74^ The XFM–CT reconstructions through the stem (Fig. 9) revealed that Ni is mainly concentrated in the apoplastic space surrounding epidermal cells, and in some epidermal cell vacuoles.^31^ The localization in the apoplastic space is likely to be deported into the epidermal cells through commonly used destructive sample preparation techniques, unless full cryo-techniques were done here. The Compton scatter map clearly reveal the sclerenchyma rays that provide structural support, epidermal cells surrounding the spongy mesophyll and the vacuolar bundles and xylem in the pith. These structures are all extremely delicate and would likely not survive physical sectioning.

## Conclusion and outlook

XFM–CT enables “virtual sectioning” of a sample, thereby entirely avoiding all of the artefacts arising from destructive sample preparation. Moreover, the method can be used on frozen-hydrated samples, as such preserving “life-like” conditions including the chemical state of the target element, which may be probed in concert with XFM with X-ray absorption spectroscopy.^5^ Another major challenge is to avoid the destructive effects of radiation damage during synchrotron XFM. Generations of reactive chemical species are responsible for breakdown of localized organic structures, such as cells, and consequently elemental redistribution.^30^ Specimens that are in frozen-hydrated state are extremely radiation hard as the entrained water is solid thereby limiting induced analyte movement.^30^ As such, it is highly recommended to keep specimens in cryogenic state (with a cryostream or another device to cool the specimen) during XFM–CT as it will effectively preserve the specimen's integrity indefinitely.

The most important limitation is the size domain that can be integrated by XFM–CT, and to a large extent this limitation is based on fundamental physics due to attenuation of the fluorescent X-rays resulting in reconstruction artefacts. This is highly element-specific, but imminent improvements in correction algorithms will expand the size of the samples that can be interrogated. Therefore, the technique is best applied to relatively small objects, although this is element dependent (with elements of interest such As and Se allowing for plant organs up to ∼2 mm diameter). This is not necessarily an important limitation, as most plant organs of interest, such as petioles and roots, are often within this size domain, and hence accessible. Synchrotron X-ray phase-contrast imaging (PCI) tomography enables to obtain high-resolution of the 3D morphology of a specimen with exquisite detail on cellular structure.^75,76^ The structural information of the plant specimen from PCI can then be correlated with the elemental information from XFM–CT to precisely visualize localization of elements within anatomical features.

Data acquisition in synchrotron XFM–CT of millileter-sized samples are typically detector saturation limited, not flux limited, and therefore, future technological improvements in detector electronic and multi-element SDD arrays rather than the current Maia with silicon PIN (p-type–intrinsic n-type–n-type) detector elements will dramatically increase throughput, and thus decrease the acquisition time for XFM–CT. Faster throughput will not only enable more samples to be investigated within the highly restrictive timeslots available at synchrotrons, but also enable XANES XFM–CT (Mijovilovich *et al.*^5^). This approach would make it possible to obtain 3D models of chemical speciation within intact plant tissues and cells. The range of applications is limitless within the plant sciences and the study of the metallome. The case studies presented here on hyperaccumulator plants of various metal(loids) illustrate the versatility of XFM–CT as a technique to elucidate the internal distribution of target elements in plant organs. We hope that these examples will stimulate interest in this technique in the plant science community to use XFM–CT in their research.

## Supplementary Material

mfac069_Supplemental_File

## Data Availability

The data underlying this article will be shared on reasonable request to the corresponding author.
