# Supplementary figures and images for: Synchrotron XFM tomography for elucidating metals and metalloids in hyperaccumulator plants
Source: Metallomics. 2022 Sep 13;14(11):mfac069. doi: 10.1093/mtomcs/mfac069 (PMC9683111; doi:10.1093/mtomcs/mfac069)

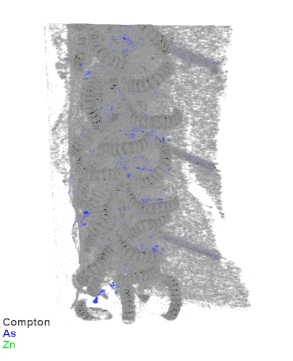

Supplement: mfac069_Supplemental_File [file mfac069_supplemental_file.zip › SI_Fig1_sporophyte_tomo1.gif]
